# Golden Pheasants and Clerical Guns

**Published:** 1890-06-07

**Authors:** 


### THE DOCTOR'S PULPIT.

### GOLDEN PHEASANTS AND CLERICAL GUNS.

A VERY gallant cavalier was the late Canon Miller when he dubbed certain members of his congregation "Golden Pheasants." Very promptly and graciously, too, did those select ones respond to his happy conceit and assume the rôle of the magnificent bird as naturally as if to the manner born. The Golden Pheasant has long been a bird of good omen at the Hospital Sunday Fund; and during the last few years he has been discovered in ever-increasing numbers in certain metropolitan congregations. It is believed that the British feathered king abounds in many parts of London, more particularly in the West, the South, and the North. But it is also held by those who know that many clerical sportsmen have entirely failed to discover him in their congregations, much less to bring him down. Nevertheless, that he is to be discovered and brought down is very certain.

Will the clergy discuss with us briefly this little problem in philanthropic sportsmanship? A pheasant is a pheasant, particularly if he is of the golden variety, and must be dealt after the manner of pheasants. It is true we have not yet succeeded in rearing him under the domestic hen, and bringing him up so tameiy that we may, like *Punch's* Frenchman, shoot him as he runs, or even after he has "stopped." Our philanthropic Golden Pheasant is still a wild and gorgeous bird of the woods, and must be approached accordingly, if he is to be bagged securely.

We cry our clerical readers' pardon for indulging in these sporting metaphors in our "Pulpit." But then they must remember that it is only a Doctor's Pulpit. Moreover, Mr. Andrew Lang, that Gorgon of modern critics, has solemnly assured the world within the last few weeks that he who wishes to be read must above all things be "light." Light enough Mr. Andrew Lang is in all conscience; but it cannot be denied that he is read; and we ourselves admit that we read him with a fearsome and self-reproving kind of pleasure.

But to return to Canon Miller and his Golden Pheasants. The Canon knew his congregation, and especially the richer members of it, very well. He understood human nature, too. He had found out in the course of his experience that nothing so individualises a man, and makes him realise his own importance, as the possession of wealth. Your rich man declines to be classed indiscriminately with Dick, Tom, and Harry. He is a person by himself, and demands to be treated as a distinct and separate individuality. There is nothing either unnatural, or immoral, or even foolish in this. Dick, Tom, and Harr[illegible] if they were rich, would do exactly the same. T[illegible]erefore, when Canon Miller had an object to serve in which the richer members of his congregation could help him much more than the poorer ones, he set about his work with the skill of an experienced commander. He knew that merely to aim a broadside at them from the pulpit, however well aimed and heavy that broadside might be, would only result in bringing down a certain portion of his rich Golden Pheasants. His firm intention was to bring them all down, even to the very last rooster: and, in order to do this, he found that the only successful plan was to take them "one by one."

Canon Miller's plan was perfectly simple; but it was also perfectly thorough. He either wrote with his own hand a special letter to each wealthy and moderately wealthy person in his congregation, or he prepared a carefully worded circular and addressed it with his own hand. That circular he called the "Golden Pheasant" circular, and had it sent to every man and woman in his congregation who could lay any sort of claim to kinship with feathered royalty.

As the clergy are necessarily very practical persons in all matters relating to collections, they would no doubt like to know the results of this particular plan. We cannot give the exact figures of Canon Miller's collections, though he found his method entirely successful; but Canon Fleming, at Chester Square, and Mr. Forrest, of St. Jude's, Kensington, have both adopted the Golden Pheasant method, and with the most striking success. Five or six years ago the collection at St. Michael's, Chester Square, for the Sunday Fund, amounted to no more than £500. Canon Fleming was well convinced that his congregation were both able and willing to give twice that amount if they were properly approached. He, therefore, commenced the systematic method of philanthropic sportsmanship which he has continued ever since, and which has resulted in raising the contributions at Chester Square from £500 to more than £1,000 a-year. Canon Fleming's plan is this: About six weeks before Hospital Sunday he begins to devote half-an-hour every day to the writing of autograph letters to the wealthy members of his congregation; and he does not cease until every man and woman, whether actually then present in London, or visiting the country, or travelling for health, pleasure, or business, in any part of the British Empire, or the world, has received a personal letter, written and addressed by the Canon's own hand, reminding him that Hospital Sunday is at hand, and asking for the usual, or, if possible, an increased contribution. The result of these earnest and strenuous efforts is known to the world.

Still more striking, though perhaps less generally known, is the case of St. Jude's, Kensington. Two or three years ago the collection at that church amounted to no more than £280. The vicar, the Rev. Mr. Forrest, was convinced that much more than that sum was possible to his congregation. He, too, followed on Canon Miller's lines, improving his methods as Canon Fleming had done. Last year, instead of £280, the magnificent sum of £1,200 was collected.

Now, this is business. The Lord Mayor, himself a man of business, sees very clearly that if like methods were universally adopted, like results would universally be secured. He has written to all the papers a brief and pithy letter on these lines. His call to the churches and chapels is for double the amount raised last year. That amount was nearly £42,000. THE HOSPITAL is now, and from its birth has been, bolder than even the Lord Mayor. £100,000 is the minimum amount which represents the increased necessities of the metropolitan hospitals; £100,000, therefore, THE HOSPITAL has inscribed on its banner; and it will not be content, or cease from its efforts, until the last guinea of the £100,000 is safe in the treasury of the Sunday Fund. Since 1885, the year before that from which THE

Hospital dates its birth, the fund has increased more than £7,000. In 1885 it amounted to £34,320, whilst the total in 1889 was £41,744.

It is as plain as Ben Lomond from the Loch that the clergy themselves have it in their power to more than double the fund. The examples of Canon Fleming and the Vicar of St. Jude's are proof positive of this. There are many congregations both in the Church and in Nonconformity who see their preachers destitute of enthusiasm, who hear nothing but the same stale platitudes, and the same feeble and sickly appeals to mere sentiment that they have been familiar with for the last dozen years. No great results can flow from such advocacy as this. Is the Christian faith worthy of being proclaimed with the intensest zeal and the most convincing logic of which any man is capable? And is the Christian care of the sick poor one of the most obvious and convincing excellencies of the Christian faith? Should not, then, every defender of the faith throw himself with self-abandoning loyalty into the great work of making adequate provision for the care of the sick poor? We appeal to the clergy to consider the question again from its beginning, and to lay to heart the striking examples of Canon Fleming and the Vicar of St. Jude's. It cannot be doubted for a moment that if every clergyman and minister were to plough with Canon Fleming's heifer they would reap his abundant crop.

